# Identification of Novel Regulators of the JAK/STAT Signaling Pathway that Control Border Cell Migration in the *Drosophila* Ovary

**DOI:** 10.1534/g3.116.028100

**Published:** 2016-05-11

**Authors:** Afsoon Saadin, Michelle Starz-Gaiano

**Affiliations:** Department of Biological Sciences, University of Maryland Baltimore County, Maryland 21250

**Keywords:** *Drosophila*, JAK/STAT regulation, cell migration

## Abstract

The Janus Kinase/Signal Transducer and Activator of Transcription (JAK/STAT) signaling pathway is an essential regulator of cell migration both in mammals and fruit flies. Cell migration is required for normal embryonic development and immune response but can also lead to detrimental outcomes, such as tumor metastasis. A cluster of cells termed “border cells” in the *Drosophila* ovary provides an excellent example of a collective cell migration, in which two different cell types coordinate their movements. Border cells arise within the follicular epithelium and are required to invade the neighboring cells and migrate to the oocyte to contribute to a fertilizable egg. Multiple components of the STAT signaling pathway are required during border cell specification and migration; however, the functions and identities of other potential regulators of the pathway during these processes are not yet known. To find new components of the pathway that govern cell invasiveness, we knocked down 48 predicted STAT modulators using RNAi expression in follicle cells, and assayed defective cell movement. We have shown that seven of these regulators are involved in either border cell specification or migration. Examination of the epistatic relationship between candidate genes and *Stat92E* reveals that the products of two genes, *Protein tyrosine phosphatase 61F* (*Ptp61F*) and *brahma* (*brm*), interact with *Stat92E* during both border cell specification and migration.

Cell migration is a fundamental and precisely regulated biological process. Although it is essential for normal embryonic development, wound healing, and immune response, cell invasion can also lead to metastasis of cancer cells (Mehlen and Puisieux 2006; Friedl and Gilmour 2009; Friedl *et al.* 2012). Hence, a comprehensive understanding of the molecular mechanisms by which invasive cells detach from an epithelial origin and gain migratory ability is of great interest for both basic and translational sciences.

The Janus Kinase/Signal Transducer and Activator of Transcription (JAK/STAT) signaling pathway is involved in the conversion of stationary epithelial cells to invasive cells, and in the regulation of their migration (Silver and Montell 2001; Silver *et al.* 2005; Hou *et al.* 2002). The requirement of the pathway for cell migration has been shown in different model organisms including zebrafish, fruit flies, and mammals (Yamashita *et al.* 2002; Naora and Montell 2005; Kira *et al.* 2002; Sano *et al.* 1999; Melchionna *et al.* 2012). In the canonical pathway, JAK/STAT signaling becomes active upon binding of an extracellular ligand to a transmembrane receptor that is constitutively associated with JAK (Kisseleva *et al.* 2002). Ligand binding causes dimerization and consequently transphosphorylation of the receptors by the associated JAKs. The phosphorylated receptor recruits STAT, which binds to a phosphotyrosine and becomes phosphorylated by JAK. Phosphorylated STAT dimerizes and moves to the nucleus to regulate transcription of downstream target genes. In contrast to the multiple JAK/STAT pathway components in vertebrates, there is only one JAK (encoded by the gene *hopscotch*), one STAT (encoded by *Stat92E*), three ligands, and one receptor in *Drosophila*. This simplicity, along with the amenability of flies for genetic manipulations and the achievement of live cell imaging *in vivo*, makes the fruit fly egg chamber an outstanding model for investigating the mechanism by which the JAK/STAT pathway regulates cell migration (Prasad *et al.* 2007; Hudson and Cooley 2014; Chen *et al.* 2014; Manning and Starz-Gaiano 2015).

Different cell types in the *Drosophila* ovary acquire migratory characteristics during oogenesis (Dobens and Raftery 2000; Horne-Badovinac and Bilder 2005). The ovary is composed of strings of ovarioles, and each string is composed of egg chambers at different developmental stages (Bate and Martinez Arias 1993; Montell 2003). Each egg chamber contains 15 large nurse cells and an oocyte, which are enveloped by a layer of about 1000 follicle cells (McLean and Cooley 2014). Early in oogenesis, a pair of follicle cells at the anterior and posterior ends of the egg chamber becomes differentiated into “polar cells”. Restriction of this fate to only two cells depends on JAK/STAT signaling (Borensztejn *et al.* 2013). Unpaired (Upd), an extracellular ligand secreted by the polar cells, activates the JAK/STAT pathway in about four to eight neighboring follicle cells in stage 8 egg chambers, which induces specification of the “border cells” (Silver and Montell 2001; Ghiglione *et al.* 2002; Beccari *et al.* 2002; McGregor *et al.* 2002; Montell *et al.* 2012). Starting at stage 9 of egg chamber development, the border cells wrap around the nonmotile polar cells and create a cluster of migratory cells that detach from the epithelium, invade between nurse cells, and migrate toward the oocyte. This migratory cell collective is reminiscent of some types of tumor metastases (Friedl *et al.* 2012). At stage 10, the border cell cluster reaches the border of the oocyte. JAK/STAT signaling is essential for both specification and migration of the cluster (Silver and Montell 2001; Beccari *et al.* 2002; Silver *et al.* 2005). STAT regulates transcription of different genes including a transcription factor, *slow border cells* (*slbo*), in the egg chamber (Beccari *et al.* 2002; Montell *et al.* 1992). Microarray analyses suggest that Slbo regulates genes involved in cell-cell adhesion, cytoskeletal arrangement, vesicle trafficking, and microtubule dynamics during border cell migration (Wang *et al.* 2006; Borghese *et al.* 2006).

A number of studies suggest that *Drosophila* STAT (Stat92E) has various regulators in different tissues (Starz-Gaiano *et al.* 2008; Yoon *et al.* 2011; Kallio *et al.* 2010; Aranjuez *et al.* 2012; Lin *et al.* 2014; Vidal *et al.* 2010). To identify regulators of this signaling pathway at the genomic scale, scientists have taken advantage of RNA interference (RNAi) technology, which disrupts gene expression at the mRNA level (Perrimon *et al.* 2010). Genome-wide RNAi analyses using STAT-activated Luciferase reporter assays in cultured *Drosophila* cell lines have indicated that the JAK/STAT pathway could have more than 100 regulators (Baeg *et al.* 2005; Müller *et al.* 2005). However, these studies yielded many different results (Müller *et al.* 2008), suggesting a need to examine context-specific STAT regulation. Some predicted regulators of the pathway, including Unpaired, Domeless, Apontic, and Socs36E, have well-characterized functions in border cell migration (Silver and Montell 2001; Silver *et al.* 2005; Beccari *et al.* 2002; Ghiglione *et al.* 2002; Starz-Gaiano *et al.* 2008, 2009; Monahan and Starz-Gaiano 2013). Either excessive or insufficient STAT activity leads to border cell specification and/or motility defects (Silver and Montell 2001; Yoon *et al.* 2011; Starz-Gaiano *et al.* 2008). Here we have performed an *in vivo*, tissue-specific RNAi-mediated reduction of a subset of putative regulators to find novel modulators of STAT activity that control cell invasion. From these candidates, we found new roles for several genes, including *Protein tyrosine phosphatase 61F* (*Ptp61F*), and *brahma* (*brm*), in the regulation of border cell specification and migration. Our results support the idea that the requirement for STAT regulators varies in different cell types to maintain precise signaling levels.

## Materials and Methods

### Fly stocks

Transgenic RNAi fly lines were obtained from the Vienna *Drosophila* RNAi Center and Bloomington *Drosophila* Stock Center and are listed in Table 1 and Supplemental Material, Table S1. All other flies were acquired from the Bloomington Stock Center, including: UAS-mCD8-GFP/CyO (Lee and Luo 1999), the anterior follicle cell drivers: c306-Gal4 (Manseau *et al.* 1997) and *slbo*-Gal4 (Rørth *et al.* 1998), the eye driver: Gal4-*ey* (Hauck *et al.* 1999), the heat shock fly line for qRT-PCR experiments: Hsp70-Gal4 (Brand and Perrimon 1993), and fly lines used for overexpression experiments: UAS-*hop^Tum-l^* (Harrison *et al.* 1995), UAS-*brm* (Stefan Thor, personal communication to FlyBase), and UAS-*Ptp61F*/TM6C, Sb^1^ (Baeg *et al.* 2005).

### In vivo RNAi knock down and overexpression

Virgin c306-Gal4 female flies were crossed to males from each UAS-RNAi line or UAS-*brm* and UAS-*Ptp61F* lines. The flies were cultured at 25°. In cases where the offspring were not viable, the crosses were kept at 18°. The newly eclosed adult females were incubated on yeast supplemented food at 29° for 14 hr for efficient Gal4-dependent expression. Ovaries from young females (less than a week old) were dissected, fixed, and stained following the protocol in the section *Antibodies*, *immunostaining*, *and microscopy*, and stage 10 egg chambers were scored for specification and/or migration defects of the border cell cluster. In this study, egg chambers with border cell specification defects were characterized as the ones containing either extra or no invasive cells when all cells were immunolabeled and stained for nuclear markers, and egg chambers with border cell migration defects were defined as those in which the border cell cluster did not reach the oocyte by the end of stage 10 (incomplete migration). UAS-*mCherry*-RNAi and UAS-*Rab5* RNAi (Assaker *et al.* 2010) were used as negative and positive controls for the RNAi analyses, respectively.

To knock down *brm* in the eye, virgin *ey*-Gal4 female flies were crossed to UAS-*brm* RNAi males and cultured at 25°. After 6 d, the larvae were moved to 29° for 2 d before they were transferred back to 25° to complete their development.

### Antibodies, immunostaining, and microscopy

Antibodies and the working dilutions were as follows: mouse anti-Armadillo (Arm) 1:40 (N2 7A1, DSHB) (Riggleman *et al.* 1990), mouse anti-Eya 1: 100 (10H6, DSHB) (Bonini *et al.* 1993), rabbit anti-STAT 1:100 (provided by Dr. D. Montell; Jang *et al.* 2009), rabbit anti-Apt 1:1000 (provided by S. Hirose; Liu *et al.* 2003), rat anti-Slbo 1:1000 (provided by Dr. P. Rorth; Beccari *et al.* 2002), and anti-rabbit GFP 1:250 (Life Technologies/Invitrogen). Secondary antibodies were Alexa Fluor 488 and 568 (Life Technologies/Invitrogen) 1:400. Ovaries were dissected to ovarioles in Schneider’s media and fixed in 4% paraformaldehyde in 0.1 M potassium phosphate buffer (McDonald *et al.* 2006). Fixed ovarioles were washed in NP40 wash buffer [0.05 M Tris HCl, pH 7.4, 0.15 M NaCl, 0.5% Nonidet P-40 (Igepal CA-630, Sigma-Aldrich), 1 mg/ml BSA, 0.02% sodium azide] (McDonald *et al.* 2006), and immunostained with α-Armadillo antibody following a previously described protocol (McDonald *et al.* 2006). Briefly, the ovarioles were incubated in primary antibody diluted in NP40 wash buffer for 3 hr at room temperature followed by four washes and secondary antibody staining overnight at 4°. The immunostained egg chambers were then stained for 10 min with DAPI 1:1000 (Invitrogen: D1306) for nuclei visualization, washed, and mounted in 70% glycerol solution. Since Arm is highly expressed in the border cell cluster and enriched in polar cells, we primarily used antibodies against this protein to detect border cell specification or migration defects. All images were taken using a Carl Zeiss AxioImager Z1 and Apotome optical sectioning with AxioVision acquisition software. Figure assembly, image cropping, and scaling were performed using Photoshop by CS6 Adobe.

### Quantitative real-time PCR analysis of RNAi-mediated gene depletion

Gal4-Hsp 70 virgin female flies were crossed to UAS-RNAi males. The adult offspring were heat shocked for 45 min at 37°, three times a day at 1-hr intervals, for 2 d. Only female offspring of the cross with UAS-*Ptp61F* RNAi and UAS-*CG8636* RNAi and male offspring of UAS-*Fer3hch* and UAS-*mib2* RNAi were used in this experiment. The RNA was extracted from 5 to 15 heat-shocked offspring using a Qiagen RNeasy mini kit. DNase I digestion (Fermentas) followed by cDNA synthesis (BioRad iScript) was performed using 1 µg of the purified RNA. qRT-PCR was implemented using 300 ng cDNA, 2 µl primer mix (10 µM), and 10 µl iTaq Universal SYBR Green Supermix (BioRad) in a 20 µl reaction. The qRT-PCR experiments were carried out on three biological replicates, each in technical triplicates. Heat shock-driven *mCherry* RNAi flies were used as a control. *Ribosomal protein L32* (*rp49*) was used as a reference gene. Primers for *Ptp61F*, *Fer3hch*, and *mib2*, listed in Table S2, were designed according to the fly primer bank (Hu *et al.* 2013) (http://www.flyrnai.org/flyprimerbank). Primers used for *rp49* were: Forward, GTGAAGAAGCGCACCAAGCAC, Reverse, ACGCACTCTGTTGTCGATACCC. Primers for *CG8636* were: Forward, AATCAGAATGCCGGGCGTTGA, Reverse, TCACGTACTTCTGTCCGTTCT.

### Quantification of Stat92E staining intensity

A master mix of 1:100 diluted anti-Stat92E antibody was used to ensure all samples contained the same amount of the antibody. Egg chambers of both the wild-type and knock down flies were fixed and stained on the same day using a 100 μl aliquot of the diluted antibody. Anti-Armadillo antibody was added to each experimental tube as a positive immunostaining control. Secondary antibody and DAPI DNA dye were also introduced to egg chambers of each genotype after being diluted in a master mix. Images of different stage egg chambers of wild type and mutant genotypes were captured with the same exposure time for each staining. A line was drawn over each visible border cell nucleus in the cluster by the “Measurement and Annotation” tool in Zeiss AxioVision software. This process was done for the same number of wild-type and mutant egg chambers. The STAT and DAPI signal intensity of each line was quantified by pixel intensity. The average STAT intensity of each nucleus was divided by the average intensity of DAPI in the same cell to normalize for differences in staining or brightness due to the focal plane/tissue depth. The average intensity per genotype was calculated using Microsoft Excel. The average STAT/DAPI staining ratio of the cells in the wild-type egg chambers was calculated and compared to that in the mutant egg chambers.

### Data availability

The authors state that all data necessary for confirming the conclusions presented in the article are represented fully within the article.

## Results

### Ovary-specific RNAi depletion of putative STAT signaling modulators

Genome-wide RNAi analyses using STAT-activated Luciferase reporters in cultured *Drosophila* cell lines have identified many regulators of STAT signaling (Baeg *et al.* 2005; Müller *et al.* 2005). Each of the two studies identified more than 100 components as regulators of the pathway, but only 20 components overlapped between the results (reviewed in Müller *et al.* 2008). Using the Gal4-UAS system (Brand and Perrimon 1993) to implement *in vivo* RNAi (Perrimon *et al.* 2010), we examined three classes of the predicted STAT modulators: those components in common between the two data sets, those with the most dramatic, validated effects described by Baeg *et al.* (Baeg *et al.* 2005) (STAT transcriptional activity changed over a threshold 2× more or 0.4 × less), and those identified by Muller *et al.* (Müller *et al.* 2005) with a known human disease gene homolog (Table 1 and Table S1). We assayed these putative regulators for their cell-autonomous effects on border cell migration.

**Table 1:** The top seven novel regulators of border cell specification/migration identified in this study

Name of the Candidate Gene	Predicted Effect on STAT Activity in Cell Culture	Screened RNAi Lines	Penetrance of the Phenotype Caused by the RNAi, %
**CG8636** (translation initiation factor)	Positive (Baeg *et al.* 2005)	v28937	Not viable
		v105325	62.35
		GLC01430	2.20
***Ptp61F*** (*Protein tyrosine phosphatase*)	Negative (Baeg *et al.* 2005)	HMS00421	48.30
	Negative (Müller *et al.* 2008)	v37436	40.50
		v37437	47.70
**α*-Snap*** (*soluble NSF* *attachment protein*)	Positive (Baeg *et al.* 2005)	v101341	Not viable
		JF03266	46.60
		v22379	38.50
		HMS00872	None
		HM04019	31
***brm*** (*brahma*)	Negative (Baeg *et al.* 2005)	v37720	14.85
	Positive (Müller *et al.* 2008)	v37721	15.80
		GL00090	None
		HMS00050	None
***Fer3HCH*** (*ferritin 3* *heavy chain homolog*)		v40505	30.70
	Positive (Müller *et al.* 2008)	HMC03397	None
***mib2*** (*mind bomb 2*)	Negative (Müller *et al.* 2008)	v40079	26.75
***CG12484*** *(immunoglobulin superfamily)*	Positive (Baeg *et al.* 2005)	v25576	18.60
		v104814	2.30

Listed based on the severity of the phenotype using a c306-Gal4 driver, with the strongest candidate (the highest penetrance) at the top. For novel genes, the predicted functions or conserved domains are given in parentheses.

**Figure 1:**
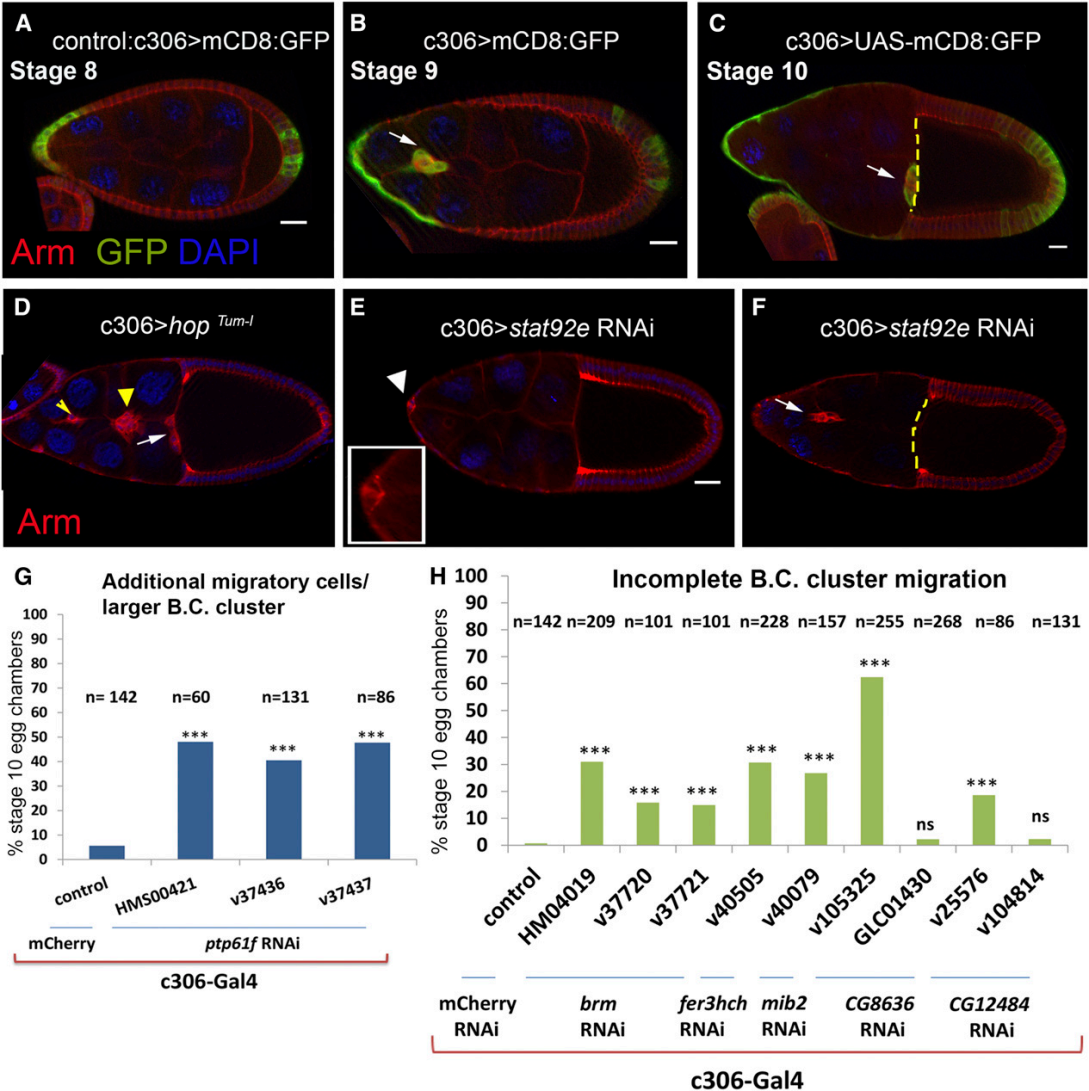
An *in vivo* RNAi screen identifies new regulators of the JAK/STAT signaling pathway that control border cell specification and migration. c306-Gal4 drives membrane-tethered GFP expression in the anterior follicle cells including border cells, prior to migration at stage 8 (A), and during normal migration at stages 9 (B) and 10 (C). For all panels, anterior is to the left and arrows indicate the border cell cluster. Yellow dashed lines indicate the oocyte border. GFP (green) shows the domain of Gal4-mediated expression of the candidate RNAs in the screen, red indicates a component of cell adhesion complex, Armadillo, and blue is DAPI, which stains the nuclei. (D) Expression of a constitutively active mutant allele of *hop* (*hop*^Tum-l^) in the anterior follicle cells leads to formation of additional migratory cells, indicated by yellow arrowhead. Expression of *Stat92E* RNAi either disrupts motile cell specification (E) or migration (F). White arrowhead and the magnified view inset in (E) indicate the polar cells. (G) RNAi knock down of *Ptp61F* in the anterior follicle cells causes formation of bigger cluster/additional migratory cells. All three RNAi lines yield in the same phenotype with similar penetrance. (H) RNAi knock down of *brm*, *Fer3hch*, *mib2*, *CG12484*, and *CG8636* by c306-Gal4 crossed to the indicated RNAi lines results in incomplete migration of the border cell cluster. The result for *brm* RNAi (HM04019) is the average of five independent experiments. The results for *Fer3hch*, *mib2*, and *CG8636* (v105325) are each the average of two independent experiments. *mCherry* RNAi is a control. B.C. in graphs abbreviates border cell. Two-tailed Fisher’s exact test was used for statistical analyses (*** *P* < 0.0005; n.s., not significant). Scale bars are 20 µm.

In total, we independently knocked down 48 predicted STAT regulators in the anterior follicle cells using c306-Gal4 (Silver *et al.* 2005; Manseau *et al.* 1997) and 80 different RNAi lines (Dietzl *et al.* 2007; Perkins *et al.* 2015). c306-Gal4 drives expression in the anterior follicle cells including the border cells by stage 8 of oogenesis, and throughout border cell migration (Figure 1, A−C). We dissected and stained ovaries from at least five F1 offspring of Gal4 females crossed to males from each of the RNAi lines. To detect border cells, we used an antibody specific for β-catenin (encoded by the *armadillo* (*arm*) gene in flies), which is expressed in follicle cells and enriched in the border cell cluster, including polar cells (Figure 1, A−C) (Peifer *et al.* 1993). We screened egg chambers for mutant phenotypes reminiscent of those caused by changes in STAT activity in the follicle cells (Figure 1, D−F). Overactivation of the pathway leads to formation of additional migratory cells, and sometimes delays cluster migration, while down-regulation results in either poor border cell specification or incomplete migration of the cluster. In addition, we knocked down the known regulator *Rab5* in the anterior follicle cells (Table S1) (Assaker *et al.* 2010) as a positive control for our approach.

With this strategy, we uncovered seven novel regulators of border cell specification/migration, summarized in Table 1. To gain insight about the temporal requirement of the identified regulators, we depleted those candidate genes in the anterior follicle cells, this time using *slbo*-Gal4 (Rørth *et al.* 1998). *slbo*–Gal4 drives expression in the anterior follicle cells at stage 9 of oogenesis, when border cells are specified, and remains active throughout migration (Montell *et al.* 1992; Rørth *et al.* 1998). Of the seven genes, only *Ptp61F* resulted in an RNAi-mediated phenotype using the *slbo*-Gal4 driver (data not shown). This suggested that later depletion of other candidates is not sufficient to cause a mutant phenotype.

### Diverse, newly identified regulators of border cell migration

Remarkably, the novel candidate regulators of border cell specification and/or migration identified in this study have a wide variety of specific cellular functions (Table 1). For instance *brm* encodes a chromatin remodeler (Tamkun *et al.* 1992), *mind bomb 2* (*mib 2*) encodes a ubiquitin ligase (Lai *et al.* 2005), *CG12484* is a member of the immunoglobulin superfamily (Vogel *et al.* 2003), and *CG8636* encodes a predicted translation initiation factor (Lasko 2000). Next, we wanted to know whether these new candidate regulators of border cell migration are involved in STAT regulation in the anterior follicle cells.

**Figure 2:**
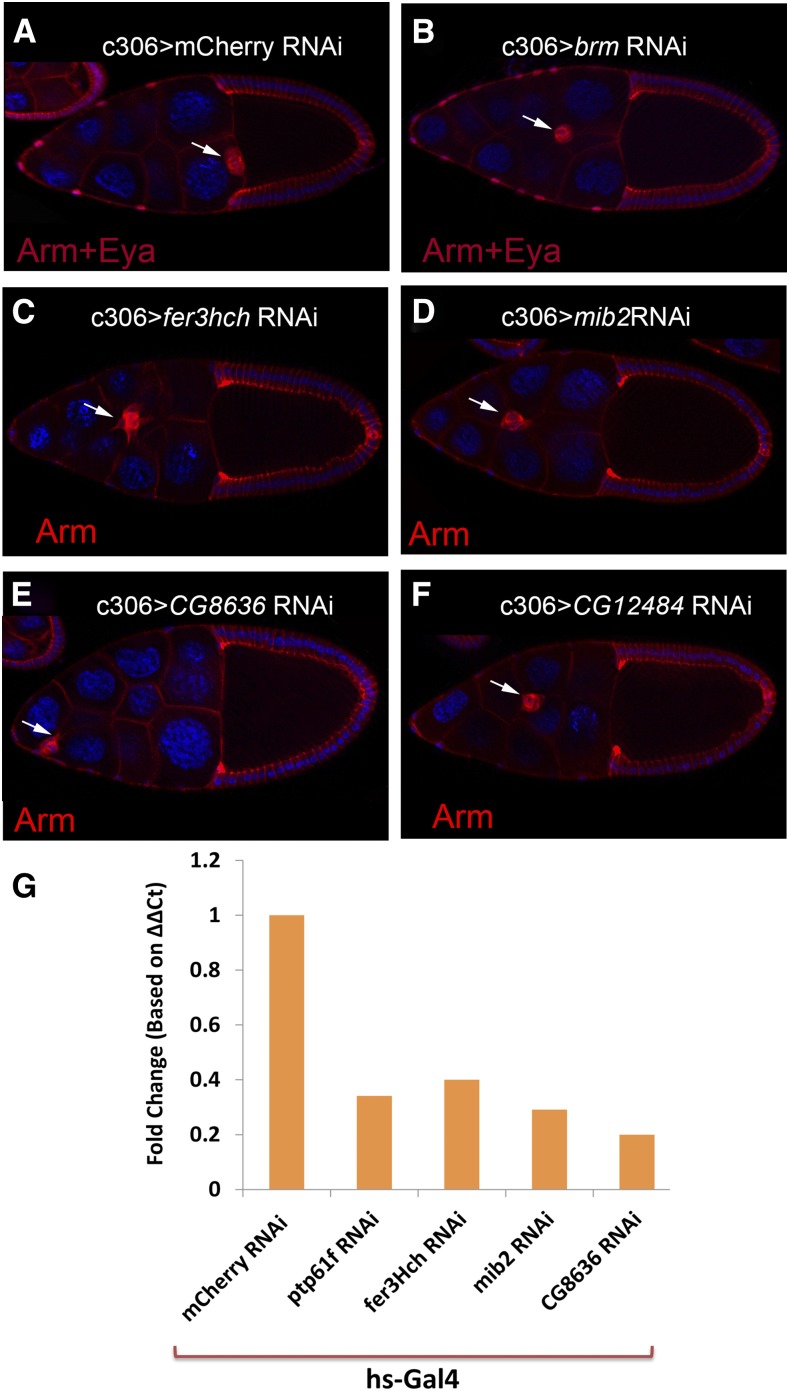
*In vivo* RNAi knock down of five candidate genes disrupts proper border cell cluster migration. Border cell cluster normally reaches to the oocyte border at stage 10, as shown here (A). Red shows Armadillo expression and blue shows DAPI. Depletion of *brm*, *Fer3hch*, *mib2*, *CG8636*, and *CG12484* in anterior follicle cells leads to incomplete migration of the border cell cluster at stage 10 (B, C, D, E, F, respectively). (G) qRT-PCR analysis indicates reduction in the level of mRNA for candidate genes upon their RNAi expression, verifying the on-target effects of the RNAi. RNAi lines used in qRT-PCR were v37436, v40505, v40079, and v105325 for *Ptp61f*, *Fer3hch*, *mib2*, and *CG8636* respectively.

**Figure 3:**
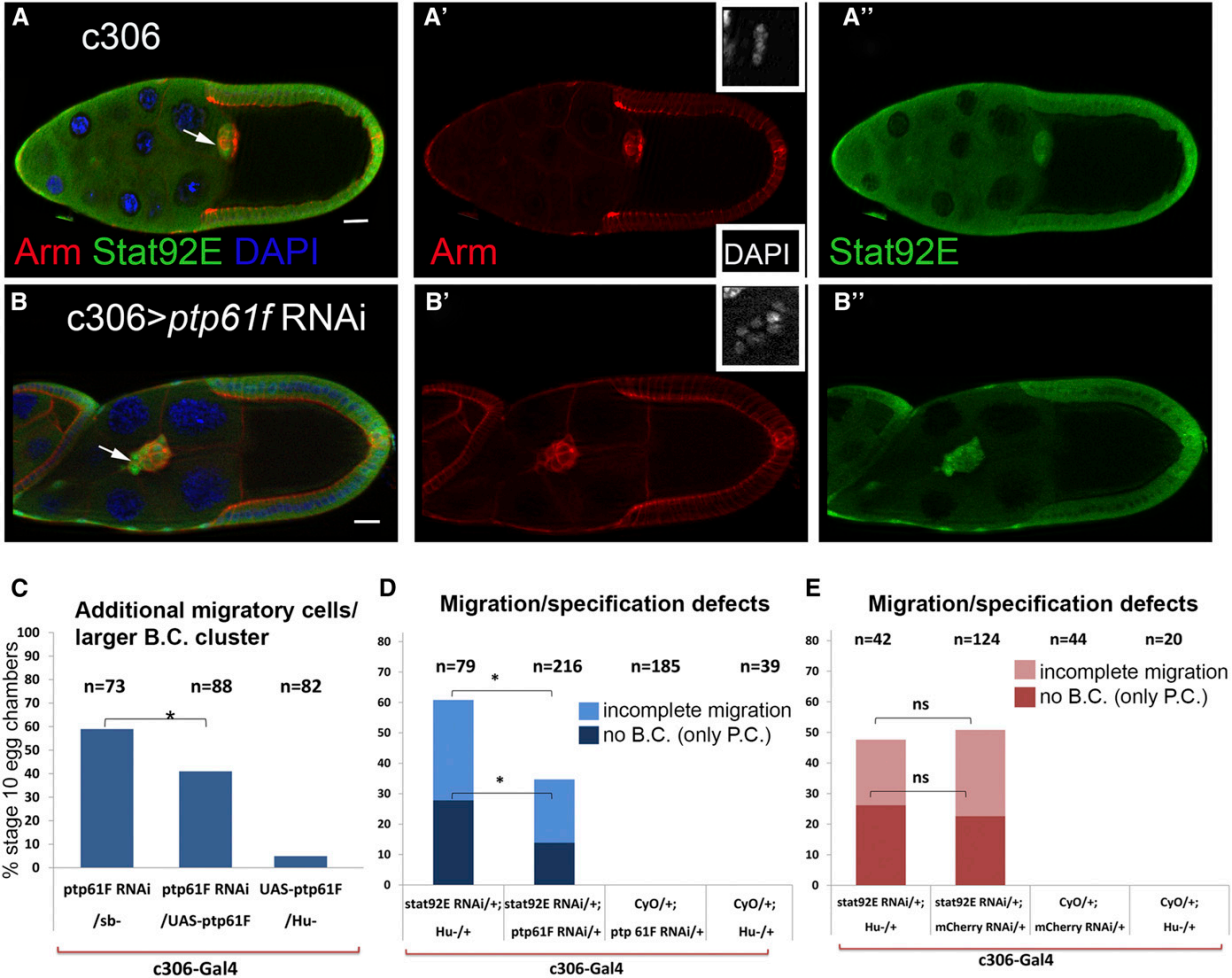
*Ptp61F* genetically interacts with *Stat92E*. Egg chambers were immunostained with antibodies for Arm (red) and Stat92E (green) proteins in control (A−A’’) and *Ptp61F* knock down (B−B’’) flies. The number of follicle cells in which STAT is activated is increased in the mutant egg chamber compared to control; this leads to formation of a bigger cluster/additional invasive cells (arrow) (B). The insets in A’ and B’ show the border cells nuclei stained with DAPI, enlarged at the same magnification. (C) Over-expression of *Ptp61F* in the anterior follicle cells rescues the additional migratory cell phenotype caused by its depletion. (D) The penetrance of the phenotypes caused by *Stat92E* depletion is compared in the single RNAi to that in the *Stat92E*, *Ptp61F* double RNAi flies. Both loss of border cells (dark blue) and incomplete migration (light blue) phenotypes caused by depletion of *Stat92E* in the anterior follicle cells are significantly suppressed in the double mutant, compared to *Stat92E* single RNAi flies. (E) *mCherry* and *Stat92E* double RNAi flies are a control for the suppression/enhancement assay. The penetrance of the phenotypes caused by *Stat92E* and *mCherry* double RNAi is not significantly different from that in the *Stat92E* single RNAi flies. Genotypes with no bars had no cases of missing border cells or incomplete migration. HMS00421 RNAi line is used to knock down *Ptp61F*. Two-tailed Fisher’s exact test was carried out to measure significance of differences (* *P* < 0.05). Scale bars are 20 µm. Arrows indicate the border cell cluster. B.C., border cell; P.C., polar cell.

Interestingly, phenotypes associated with up- and down-regulation of STAT activity both appeared in our screen (Figure 1). Among the novel candidate regulators of border cell migration, only α-*Soluble NSF attachment protein* (α*-Snap*) and *Ptp61F* altered border cell specification. Reduction of α*-Snap* in the anterior follicle cells resulted in few to no border cells, while reduction of *Ptp61F* led to the formation of bigger clusters/additional invasive cells (Figure 1G, Figure 3B, and Figure S1, C and D), similar to gain-of-function mutations in STAT and/or its activators (Figure 1D) (Silver and Montell 2001). In contrast, reduction of *brm*, *Ferritin 3 heavy chain homolog* (*Fer3hch*), *mib2*, *CG12484*, or *CG8636* gene expression in the anterior follicle cells caused incomplete migration of the cluster (Figure 1H and Figure 2, B−F), similar to the *Stat92E* loss of function phenotype (Figure 1, E and F). Our results suggested that these factors might not regulate STAT activity in follicle cells in the same fashion as predicted by the results in cultured cells. For instance, *mib2* is identified as a negative regulator of STAT activity in cultured cells; however, the phenotype caused by its depletion resembles that caused by STAT down-regulation in egg chambers.

### Validation of the RNAi-mediated knock down results

To ensure that the phenotypes observed upon RNAi knock down of the genes shown in Table 1 were due to on-target effects, we took several different approaches. Primarily we tested multiple RNAi lines, targeting at least two different parts of the gene, for each candidate (Table 1 and Figure 1, G and H). To ascertain the effectiveness of the *Ptp61F*, *mib2*, *Fer3hch*, and *CG8636* RNAi lines, we also performed qRT-PCR to analyze the alteration in the level of each message (Figure 2G). Significant reductions in the levels of each mRNA supported on-target effects. Widespread down-regulation of α*-Snap* caused lethality, so we did not pursue it in this study. Since only one strong RNAi line could viably be expressed in border cells for *mib2*, *Fer3hch*, and *CG8636* we chose not to characterize these genes any further. Instead we focused on *Ptp61F* and *brm* since they had highly penetrant effects with at least two transgenic RNAi lines and additional, available reagents for other genetic manipulations. Furthermore, potential involvement of these two genes in STAT-mediated border cell specification and migration had not been characterized previously.

**Figure 4:**
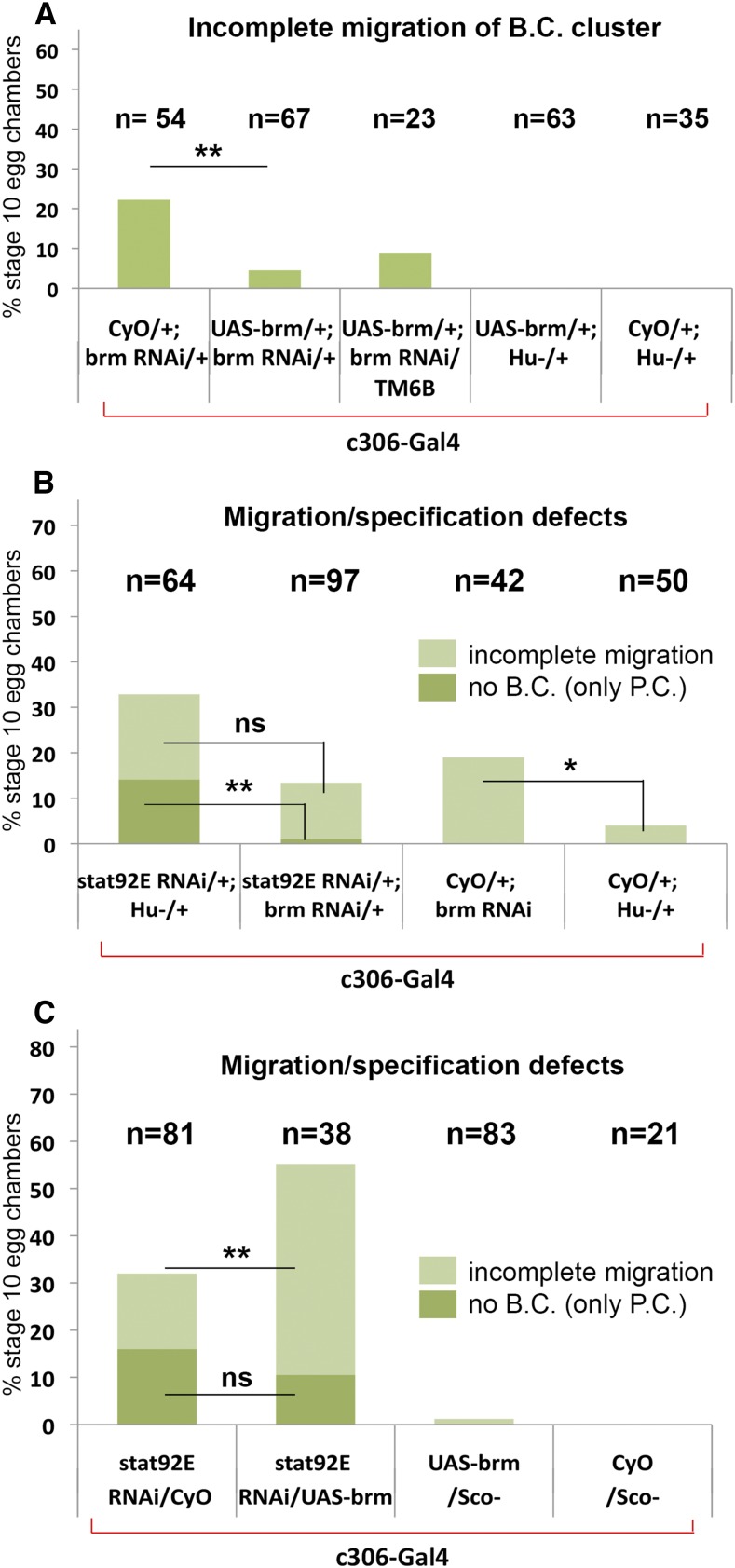
*brm* genetically interacts with *Stat92E*. (A) Anterior follicle cells overexpressing *brm* in its depleted background significantly rescue the phenotype caused by the RNAi; however, overexpression in the control background causes no phenotype. (B) Depletion of *Stat92E* and *brm* together in the anterior follicle cells significantly reduces the penetrance of border cell specification defects (dark green), but not migration defects (light green) caused by *Stat92E* RNAi. (C) Overexpression of *brm* together with depletion of *Stat92E* in the anterior follicle cells enhances the border cell migration defect caused by *Stat92E* RNAi, but does not significantly modify specification defects. Line HM04019 RNAi was used to deplete *brm*. (* *P* < 0.05; ** *P* < 0.005; ns, not significant)

Several experiments supported the idea that the RNAi lines for *Ptp61F* and *brm* caused specific, on-target effects. In the case of *Ptp61F*, all three RNAi lines used in the study resulted in the same phenotypes with similar penetrance (Table 1 and Figure 1G). Three of the five RNAi lines used for *brm* depletion led to incomplete migration of the border cell cluster with varying penetrance (Table 1 and Figure 1H). Two of the three phenotype-producing lines for each gene have the same target sequence while the other one targets a different region. To validate the RNAi results, we expressed a dominant negative allele of *brm* using c306-Gal4; however, this led to severe disruptions in follicle cell organization and the border cells could not be analyzed. Since dominant negative *brm* causes a mutant eye phenotype (Armstrong *et al.* 2005), we knocked down *brm* using *eyeless(ey)*-Gal4 (Hauck *et al.* 1999) and the RNAi line. Ninety percent of the flies displayed a strong reduction in the eye size (Figure S2), similar to the phenotype observed by Armstrong *et al.*, confirming the target specificity of the RNAi. Partial but significant rescue of the invasive cell phenotypes by 1.4-fold (*P* < 0.05) (Figure 3C) and 4.9-fold (*P* < 0.005) (Figure 4A) upon overexpression of *Ptp61F* and *brm* respectively in their depleted backgrounds also supported the validity of these knock down lines.

### Protein tyrosine phosphatase 61F (Ptp61F) genetically interacts with Stat92E in the egg chamber

*Ptp61F* is a downstream target of the JAK/STAT pathway in *Drosophila* embryos, and it encodes a negative regulator of the pathway in some adult tissues, including the eye, immune cells, and ovary (Baeg *et al.* 2005; Buszard *et al.* 2013). Thus, Ptp61F acts as part of a negative feedback loop, and it is thought to function by dephosphorylating JAK and possibly STAT (Baeg *et al.* 2005). Female flies homozygous null for *Ptp61F* have a shorter life span and reduced fecundity (Buszard *et al.* 2013). Deletion of the gene increases the level of phosphorylated Stat92E in ovary tissue homogenate, suggesting an interaction between *Ptp61F* and *Stat92E* in the ovary (Buszard *et al.* 2013).

*In vivo* RNAi knock down of *Ptp61F* in the anterior follicle cells caused the formation of a bigger border cell cluster and/or additional migratory cells in about 40–50% stage 10 egg chambers, depending on the RNAi line (Figure 1G and Figure 3B). Interestingly, in most cases these larger clusters migrated normally (Figure 3B and Figure S1, C and D). The additional invasive cells appeared in a variety of arrangements. In some scenarios all invasive cells adhered together and formed a bigger cluster (Figure 3B), while in other cases some extra cells adhered to the main cluster and some trailed behind separately (Figure S1C).

The phenotype caused by depletion of *Ptp61F* in the anterior follicle cells was similar to that caused by overactivation of STAT via the constitutively active JAK, *hop^Tum-l^* mutant (Corwin and Hanratty 1976; Harrison *et al.* 1995; Silver and Montell 2001) (Figure 1D). Since border cells are postmitotic, the additional invasive cell phenotype observed upon RNAi knock down of *Ptp61F* in the anterior follicle cells was consistent with an inhibitory effect of Ptp61F protein on STAT activity and changes in cell fates. To test this, we looked at the expression pattern of activated STAT and the gene products of two of its known downstream targets, Apontic (Apt) (Starz-Gaiano *et al.* 2008) and Slow border cells (Slbo) (Silver and Montell 2001), in egg chambers with reduced *Ptp61F* expression (Figure 3B and Figure S1, C−D). Using these and Eyes absent (Eya) (Bai and Montell 2002) expression as follicle cell markers, we detected up to 15 invasive cells, with an average of 7.5 ±0.3 cells, in *Ptp61F* knock down stage 10 egg chambers (*n* = 60). In contrast, control egg chambers contained only up to eight invasive cells with an average of 5.2 ±0.1 (*n* = 88). An increased number of follicle cells expressing activated STAT, Apt, and Slbo suggested a relationship between *Ptp61F* and *Stat92E* in the epithelium. To investigate a possible interaction, we compared the border cell specification/migration defects due to *Stat92E* knock down in anterior follicle cells (Figure 1, E and F) *vs.* those due to *Stat92E* and *Ptp61F* double knock down. Depletion of *Ptp61F* significantly suppressed both of the phenotypes caused by *Stat92E* RNAi alone, reducing border cell specification defects and the migration delays by 13.9% and 12.2% respectively (Figure 3D). As a control, we created *Stat92E* and *mCherry* double RNAi flies and analyzed suppression of the phenotypes caused by the *Stat92E* depletion. *mCherry* RNAi did not significantly affect the phenotypes caused by *Stat92E* RNAi (Figure 3E). These results suggest that Ptp61F has a specific role in restricting STAT activity during border cell specification so that the appropriate number of cells is specified.

### brm genetically interacts with Stat92E to regulate border cell specification and migration

Brm, a SWI2/SNF2 homolog, functions as a transcriptional activator and repressor in a cell-type specific manner (Tamkun *et al.* 1992; Marenda *et al.* 2004; Collins and Treisman 2000; Kwok *et al.* 2015). While Brm is a positive regulator of STAT activity during larva hematopoiesis (Remillieux-Leschelle *et al.* 2002), in cultured cells Brm has been identified both as a positive and a negative regulator of STAT signaling (Müller *et al.* 2008). It has also been shown to function along with STAT as a transcription coactivator to promote target gene expression (Panov *et al.* 2012; Vorobyeva *et al.* 2009). Although some cell motility can occur in the absence of transcription, precise control over transcriptional regulation is clearly required during developmental cell movements, including for appropriate border cell migration (Montell *et al.* 2012).

Depletion of *brm* in the anterior follicle cells caused incomplete migration in 15–30% of stage 10 egg chambers, depending on the transgenic line (Figure 1H and Figure 2B). The migration delay ranged from border cell clusters that had not detached from the epithelium to ones that had completed 80% of the migration distance. Brm depletion had no effect on the development and/or appearance of other follicle cells, including posterior epithelial cells in the c306-Gal4 expression domain. This supported the idea that *brm* functions particularly in the anterior follicle cells to control border cell specification and/or migration. To investigate a possible genetic interaction between *brm* and *Stat92E* during border cell specification and migration, we knocked down *Stat92E* in the anterior follicle cells in both a *brm* overexpressing and depleted background. *brm* depletion suppressed the border cell specification defect caused by *Stat92E* RNAi alone by 14.1-fold (Figure 4B). Furthermore, *brm* overexpression enhanced the *Stat92E* RNAi-mediated border cell migration defect by 2.8-fold (Figure 4C). These results suggest that Brm potentially inhibits STAT during both cell specification and migration.

### Depletion of brm increases the level of activated STAT in the anterior follicle cells

**Figure 5:**
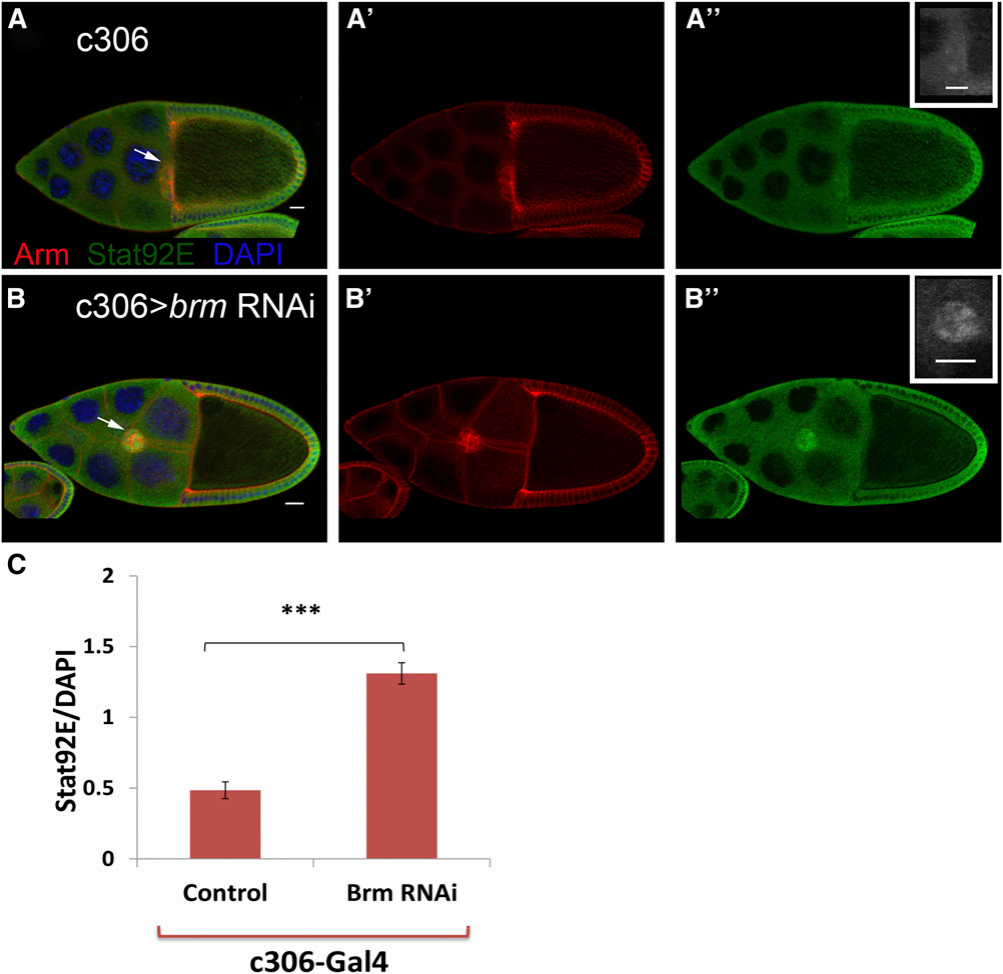
Reduction of *brm* increases the level of nuclear STAT in the anterior follicle cells. Stage 10 egg chambers are immunostained with anti-Stat92E antibody in control (A−A’’) and *brm* knock down genotypes (B−B’’). Red, green, and blue indicate Arm, STAT, and DAPI respectively. The insets in A’’ and B’’ indicate STAT immunolabeling in border cells in the control (A’’) and mutant (B’’) egg chambers. Scale bars are 20 µm. (C) The level of the nuclear STAT was quantified in 22 egg chambers of each genotype, for a total of 120 border cells in the mutant and 116 border cells in the control egg chambers. The intensity of nuclear STAT in the *brm*-depleted egg chambers is higher compared to that in controls. Two-tailed independent *t*-test was carried out (*** *P* < 0.0001).

To understand the effect of *brm* on STAT activity during border cell formation and migration further, we immunostained *brm* mutant egg chambers with anti-Stat, anti-Slbo, and anti-Apt antibodies (Figure 5 and Figure S1, E−F). We quantified the level of nuclear STAT protein in *brm* knock down and c306-Gal4 control egg chambers. Since STAT translocates to the nucleus upon activation, this can be used as a read-out for pathway activity. The level of nuclear STAT was 2.7-fold higher in the mutant background compared to the control genotype (Figure 5C), suggesting that *brm* normally down-regulates STAT activity in border cells. For this experiment, we used DAPI intensity as an imaging control; however, since Brm acts as a chromatin remodeler, DAPI signal intensity could be altered due to changes in chromatin structure. To rule out the possibility that the observed increase in nuclear STAT upon *brm* depletion is due to a reduction in the intensity of DAPI, we compared the level of this staining in the mutant cells and the controls. Interestingly, the DAPI intensity in the *brm*-depleted border cells (*n* = 120) was 1.7-fold (*P* < 0.05) higher than in the control border cells (*n* = 116). This suggests that the observed increase in activated, nuclear STAT levels in *brm*-depleted cells is underestimated. We also examined 10XSTAT92E-GFP reporter (Bach *et al.* 2007) in these genotypes. However, GFP expression was essentially saturating in border cells of control egg chambers, making it impossible to detect an increase in Brm-depleted cells. Overall, though, these results are consistent with the idea that Brm normally acts to inhibit the level/activity of STAT in the border cells.

Surprisingly there was no obvious change in the level of the STAT downstream targets Slbo or Apt in the mutants compared to control egg chambers. This suggests that Brm may function differently at several levels of the cascade to regulate the pathway during border cell specification and migration (Figure S1G).

## Discussion

Even though cell migration is required for biological events like embryonic development and immune function, it can endanger one’s life by contributing to conditions such as atherosclerosis and metastasis of cancer cells (Kraemer 2000; Mehlen and Puisieux 2006; Friedl and Gilmour 2009; Friedl *et al.* 2012). Therefore, thorough understanding of regulators of cell migration and their mechanistic effects remain crucial. In this study we took advantage of *in vivo* RNAi technology to investigate how the loss of a subset of STAT regulators identified in cultured cells (Baeg *et al.* 2005; Müller *et al.* 2005) impacts cell migration. By screening 48 candidate genes, we identified seven novel regulators of border cell specification/migration, supporting the value of cell culture assays and the power of *in vivo* RNAi. When possible, we used multiple transgenic RNAi lines, but for some, only one line was available. The result of our work (Table 1) and the studies done by other investigators indicate that some RNAi lines yield false negative results (Perrimon *et al.* 2010). Thus, it is possible that some candidate genes for which only one RNAi transgene was screened (Table S1) may still be involved in border cells but showed no phenotype due to ineffectiveness (low expression) of the RNAi. We chose to focus further experiments on two genes strongly implicated to be STAT regulators, *Ptp61F* and *brm*.

Ptp61F is a negative regulator of the JAK/STAT signaling pathway in multiple contexts, but its mechanistic effects have not been fully characterized (Baeg *et al.* 2005; Müller *et al.* 2008; Buszard *et al.* 2013). Buszard *et al.* have shown that female flies lacking *Ptp61F* have a shorter life span and reduced fecundity due to egg chamber apoptosis prior to oocyte maturation. The same study indicates that deletion of *Ptp61F* increases the level of phosphorylated STAT in the ovary (Buszard *et al.* 2013). This could suggest an interaction between Ptp61F and Stat92E that is required for fertilization. Here we showed that Ptp61F functions as a negative regulator of STAT signaling in border cells. These cells normally contribute to the formation of a structure in the eggshell called the micropyle, the sperm entry site (Montell *et al.* 1992). The bigger border cell clusters observed when *Ptp61F* is depleted (Figure 3B and Figure S1C) could in part explain the reduced fecundity previously observed (Buszard *et al.* 2013), since this might disrupt the proper formation of the micropyle. Investigation of this possibility, however, requires further research. To determine if Ptp61F triggers inactivation of the STAT signaling pathway in the cytoplasm or the nucleus of border cells, one could differentially express the cytoplasmic and the nuclear variant of the protein (McLaughlin and Dixon 1993; Buszard *et al.* 2013) in these cells. Interpretation of the results of the proposed experiment might be a challenge since, in our experience, overexpression of *Ptp61F* did not lead to a major border cell specification/migration defect (Figure 3C and data not shown). Thus, more experiments are needed to determine the key targets of Ptp61F activity during cell migration.

Brm is a core component of two multiprotein complexes (BAP and PBAP), initially identified as a member of the trithorax group (Tamkun *et al.* 1992; Kal *et al.* 2000; Mohrmann *et al.* 2004; Schuettengruber *et al.* 2011). Brm is known to activate transcription globally during *Drosophila* development by altering the chromatin structure and facilitating RNA polymerase II binding (Tamkun *et al.* 1992; Elfring *et al.* 1998; Orlando and Paro 1995; Armstrong *et al.* 2002). However, a number of studies have shown that Brm can also function in a restricted manner by either activating or repressing specific genes in particular cell types in developing flies (Marenda *et al.* 2004; Collins and Treisman 2000; Kwok *et al.* 2015). We show here that Brm is required for proper cell migration. Research in mammalian cells has suggested that the human homolog for Brm, BRG1, can regulate various cell adhesion molecules including E-cadherin (Banine *et al.* 2005; Reisman *et al.* 2009; Matsubara *et al.* 2013). To address a possible effect of Brm on cell adhesion in border cells, we examined the expression pattern/level of two well-characterized cell adhesion molecules, Armadillo and E-Cadherin (Peifer *et al.* 1993; Oda *et al.* 1997; Niewiadomska *et al.* 1999). We did not detect any obvious and consistent alteration in the level and pattern of these molecules upon RNAi knock down of *brm* (data not shown). However, a more subtle change in the expression pattern or function of Arm and E-Cad remains a possibility and may contribute to defective cell movements. We present evidence that Brm functions as a negative regulator of the JAK/STAT signaling pathway, and likely affects multiple downstream genes during border cell specification and migration.

In contrast to other negative regulators of *Stat92E* (*i.e.*, *apt*) (Starz-Gaiano *et al.* 2008), depletion of *brm* in the anterior follicle cells did not cause additional invasive cells (Figure 2B). Furthermore, despite an increase in the level of nuclear STAT, *brm* depletion did not affect the level of Slbo and Apt (Figure 5 and Figure S1, E−F). A possible explanation is that Brm could affect the STAT signaling pathway at multiple levels, in positive or negative ways. According to ModEncode data, Brm can bind to the regulatory region/s of multiple components of the STAT signaling pathway including *apt* (Roy *et al.* 2010). Apt is both a downstream target and an inhibitor of STAT activity. Therefore, it could be that Brm represses *Stat92E* while activating *apt* (Figure S1G). Even though this requires further investigation, knowing that Apt and Slbo negatively regulate each other (Starz-Gaiano *et al.* 2008) (Figure S1G) could partially explain the lack of obvious increase in the level of Apt and Slbo in the *brm*-depleted egg chambers. An additional possibility is that Brm affects signaling cascades involved in other aspects of border cell migration (*i.e.*, timing of migration) and the observed effect of *brm* depletion on border cell migration is a combinatorial effect. Investigating these as well as other possibilities will advance our knowledge in the mechanistic effect of Brm on the STAT signaling pathway during cell migration.

All novel regulators of border cell migration identified in this study have human homologs, and some are associated with disease (Hu *et al.* 2011). The two proteins most related to Ptp61F in humans are PTPN1 (also known as PTP1B) and PTPN2 (also known as PTPT) (Hu *et al.* 2011). The two most closely related Brm homologs in humans are SMARCA4 (also known as BRG1) and SMARCA2 (also known as Brm and HBRM) (Hu *et al.* 2011). Previous studies have linked up-regulation of Brm and BRG1 to metastatic ability of various cancers including pancreatic cancer, breast cancer, and melanoma (Numata *et al.* 2013; Bai *et al.* 2013; Saladi *et al.* 2010). In light of this, it will be interesting to see if Ptp and Brm family members have conserved roles in STAT-mediated cell migration in humans.

Damiano *et al.* has demonstrated that Brm functions as an inhibitor of C/EBP transcription in nonmalignant mammary epithelial cells (MCF10A MECs) (Damiano *et al.* 2014). This is very interesting because C/EBP is a transcription factor with a very well-characterized homolog in our model, Slbo (Montell *et al.* 1992). The negative effect of Brm on C/EBP further supports the functional conservation of gene products and signaling pathways between *Drosophila* and humans, suggesting the applicability of our findings in translational science. All together our findings shed light on the means by which Brm regulates cell migration and how JAK/STAT signaling is regulated in invasive cells.

## Supplementary Material

Supplemental Material
