# Supplementary material for: Identification of Novel Regulators of the JAK/STAT Signaling Pathway that Control Border Cell Migration in the Drosophila Ovary
Source: G3 (Bethesda). 2016 May 11;6(7):1991–2002. doi: 10.1534/g3.116.028100 (PMC4938652; doi:10.1534/g3.116.028100)
Supplement: Supplemental Material [file supp_6_7_1991__index.html]

Identification of Novel Regulators of the JAK/STAT Signaling Pathway that Control Border Cell Migration in the Drosophila Ovary — Supplemental Material 

# Identification of Novel Regulators of the JAK/STAT Signaling Pathway that Control Border Cell Migration in the *Drosophila* Ovary

## Supplemental Material for Saadin and Starz-Gaiano, 2016

**Files in this Data Supplement:**

- Table S1 - In cultured cell-predicted STAT regulators screened for their effect on BC specification/migration, which are not listed in Table 1. (.pdf, 216 KB)
- Table S2 - Primers from primer bank used in qRT PCR experiment. (.pdf, 44 KB)
- Figure S1 - APT and SLBO antibody staining of stage 10 driver-alone control (A-B), *ptp61f* (C-D) and *brm* (E-F) RNAi expressing egg chambers. (.pdf, 467 KB)
- Figure S2 - *brm* RNAi caused a strongly reduced eye phenotype in 90% of adult flies when expressed under the control of the *eyeless* (*ey*)-Gal4, consistent with its known function in this tissue. (.pdf, 1 MB)
- File S1 - Supplementary references. (.pdf, 57 KB)
