# Supplementary material for: Identification of Novel Regulators of the JAK/STAT Signaling Pathway that Control Border Cell Migration in the Drosophila Ovary
Source: G3 (Bethesda). 2016 May 11;6(7):1991–2002. doi: 10.1534/g3.116.028100 (PMC4938652; doi:10.1534/g3.116.028100)
Supplement: Supplemental Material [file supp_g3.116.028100_TableS1.pdf]

Table S1

(A)

Name of the Candidate Gene	Screened RNAi lines	Notes
Cdc2	GL00262	No viable offspring when crossed to c306-Gal4
	JF03004	
CG11700	HMS00726	
CG17836	HMS00053	
CG30460	v40624	
	v105307	
enok	HM05195	
Hsc70-4	GLV21049	
	JF03136	No viable offspring when crossed to c306-Gal4
ken	HMS01219	
mbl	JF03264	
mor	GL00477	
	HMS01267	No viable offspring when crossed to c306-Gal4
proct	JF03249	
Ssdp	v28608	10-15% stage 10 egg chambers had incompletely migrated BC cluster, but not followed up due to stock loss.
	v102006	

(B)

Name of the Candidate Gene	Screened RNAi lines	Notes
Ash1	HMS00582	No viable offspring when crossed to c306-Gal4
	JF01498	
**CG5546**	HMS00588	
	JF02713	
**CG5988 (Upd2)**	HMS00901	
	HMS00948	
**CG11198**	HMS01230	
CG30089	JF02953	
**CG32767**	v42023	
	v42336	
**Hbn**	JF02195	
**Hmgcr**	JF02110	
jumo	GL00363	
Nrx-1	HMS00403	
	JF02652	
Pitslre	GL00025	
trx	HMS00580	No viable offspring when crossed to c306-Gal4
	JF01557	
unc-4	HMS01603	
**ush**	HM05193	
	HMS00744	

(C)

Name of the Candidate Gene	Screened RNAi lines	Notes
**bon**	JF02373	
	HMS01657	
**Caf1**	HM04021	
	HMS00051	No viable off spring when crossed to c306-Gal4
CG10960	HMS01072	
CG11696	JF02833	
CG15784	HMS01603	30% stage 10 chambers had extra invasive cell, but homozygous deficiency failed to reproduce the same phenotype
CG31132	HMS00304	No viable off spring when crossed to c306-Gal4
CG40351	HMS00581	
CkIIα	GL00003	
	JF01436	
CtBP	HMS00677	
	JF01291	
**dre4**	GL00017	No viable off spring when crossed to c306-Gal4
	HMS01332	No viable off spring when crossed to c306-Gal4
jbug	JF01166	
mask	HMS01045	
	JF01147	
**Par-1**	GL00253	
	HMS00405	
**Rab5**	HMS00147	
	JF03335	> 90% stage 10 egg chambers had incompletely migrated BC cluster Consistent with studies by Assaker et al., 2010. Used as a (+) control
sol	JF03400	
**TSG101**	GLV21075	

Table S1- In cultured cell-predicted STAT regulators screened for their effect on BC specification/migration, which are not listed in Table 1- Listed in (A) are the candidates that were identified by both Baeg et al., and Muller et al., as STAT regulators (reviewed in Muller et al., 2008). Listed in (B) and (C) are the candidates, which were identified as STAT regulators by Baeg et al. (2005), and Muller et al., (2005) respectively. Unless otherwise noted, the listed screened RNAi lines had little to no effect on BC specification/migration. In (B) and (C) the candidate genes in the bold font are the ones that were predicted as positive regulators of STAT, and the candidates in regular font are the ones predicted as negative regulators of STAT activity.
