# Supplementary material for: Identification of Novel Regulators of the JAK/STAT Signaling Pathway that Control Border Cell Migration in the Drosophila Ovary
Source: G3 (Bethesda). 2016 May 11;6(7):1991–2002. doi: 10.1534/g3.116.028100 (PMC4938652; doi:10.1534/g3.116.028100)
Supplement: Supplemental Material [file supp_g3.116.028100_TableS2.pdf]

Gene	Forward Primer	Reverse Primer
Ptp61f	AACGGCATCGATCCAATTC	CCGCTTCAGCTCGTTCTC
Fer3hch	AGGCATCCCACCAGTATCTG	CGCTCGCCTTTAGGAAGAATC
Mib2	CTGGATCTGGTCGAATCAAGAC	CGACAGTGTTTTCTGGCGAAT

Table S2. Primers from primer bank used in qRTm PCR experiment.
