# Supplementary material for: Identification of Novel Regulators of the JAK/STAT Signaling Pathway that Control Border Cell Migration in the Drosophila Ovary
Source: G3 (Bethesda). 2016 May 11;6(7):1991–2002. doi: 10.1534/g3.116.028100 (PMC4938652; doi:10.1534/g3.116.028100)
Supplement: Supplemental Material [file supp_g3.116.028100_FigureS1.pdf]

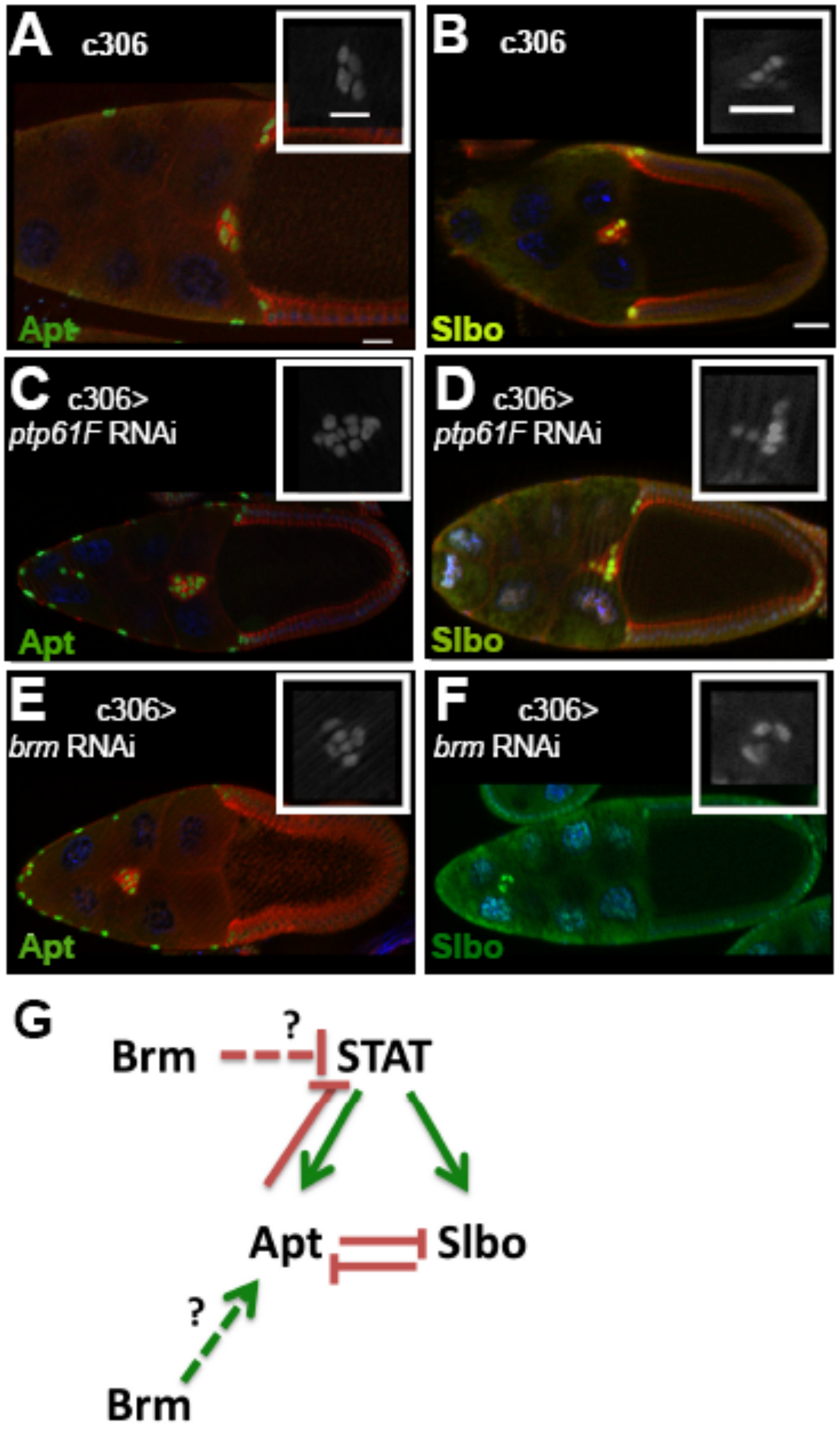

Figure S1. APT and SLBO antibody staining of stage 10 driver-alone control (A-B), *ptp61f* (C-D) and *brm* (E-F) RNAi expressing egg chambers. Red in A-E indicates ARM. Green staining corresponds to APT in A, C and E and, SLBO in B,D and F. Blue staining in all images correspond to DAPI. Insets in A, C and E are the magnified view of corresponding BC cluster stained with APT antibody. Insets in B, D and F are the magnified view of SLBO stained BC cluster. Scale bars are 20 μm.
