# Supplementary material for: Identification of Novel Regulators of the JAK/STAT Signaling Pathway that Control Border Cell Migration in the Drosophila Ovary
Source: G3 (Bethesda). 2016 May 11;6(7):1991–2002. doi: 10.1534/g3.116.028100 (PMC4938652; doi:10.1534/g3.116.028100)
Supplement: Supplemental Material [file supp_g3.116.028100_FigureS2.pdf]

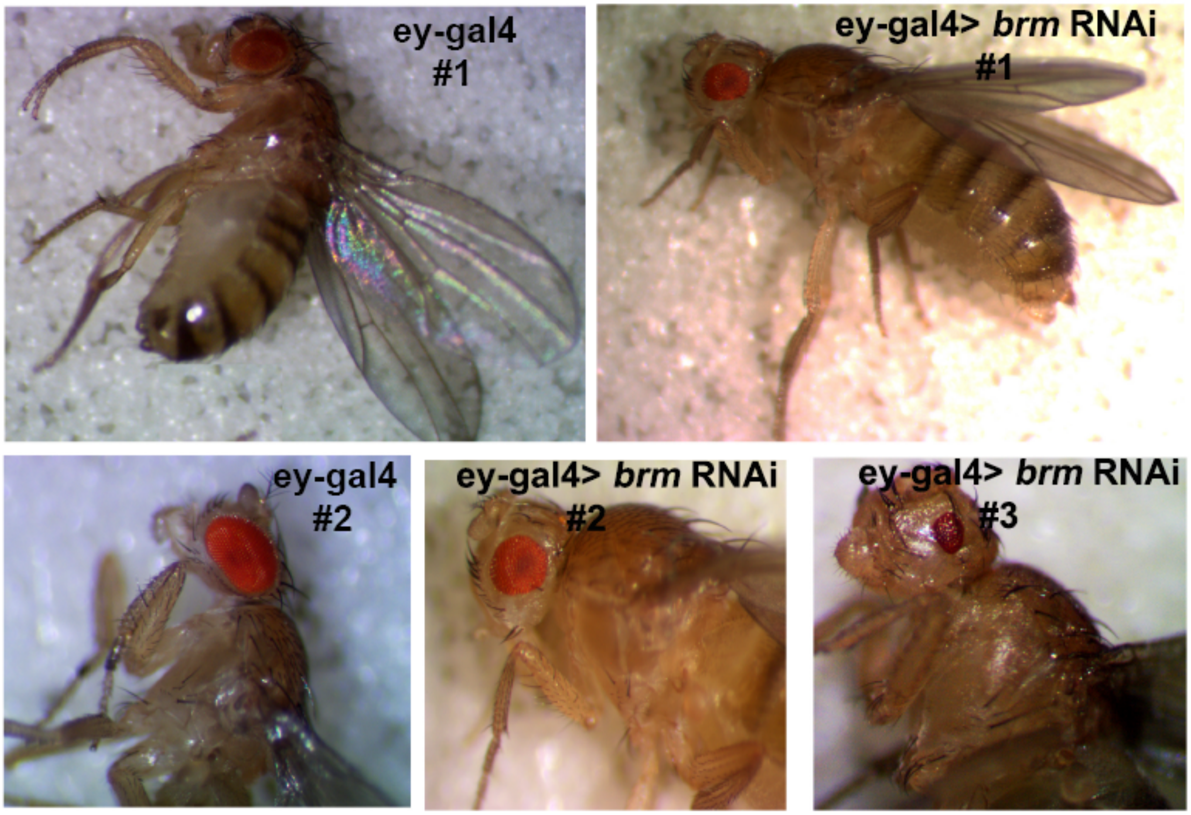

Figure S2. *brm* RNAi caused a strongly reduced eye phenotype in 90% of adult flies when expressed under the control of the *eyeless* (*ey*)-Gal4, consistent with its known function in this tissue. Line HM04019 RNAi was used to deplete *brm*.
