# Supplementary material for: Identification of Novel Regulators of the JAK/STAT Signaling Pathway that Control Border Cell Migration in the Drosophila Ovary
Source: G3 (Bethesda). 2016 May 11;6(7):1991–2002. doi: 10.1534/g3.116.028100 (PMC4938652; doi:10.1534/g3.116.028100)
Supplement: Supplemental Material [file supp_g3.116.028100_FileS1.pdf]

**Supplementary references**

Baeg, G.H., R. Zhou, and N. Perrimon, 2005 Genome-wide RNAi analysis of JAK/STAT signaling components in Drosophila. *Genes Dev* 19 (16):1861-1870.

Müller, P., M. Boutros, and M.P. Zeidler, 2008 Identification of JAK/STAT pathway regulators--insights from RNAi screens. *Semin Cell Dev Biol* 19 (4):360-369.

Müller, P., D. Kuttenkeuler, V. Gesellchen, M.P. Zeidler, and M. Boutros, 2005 Identification of JAK/STAT signalling components by genome-wide RNA interference. *Nature* 436 (7052):871-875.
